# Risk communication and community engagement capacity in the Eastern Mediterranean Region: a call for action

**DOI:** 10.1136/bmjgh-2022-008652

**Published:** 2024-02-28

**Authors:** Dahlia Samhouri, Tara Rose Aynsley, Peggy Hanna, Melinda Frost, Vicky Houssiere

**Affiliations:** 1 WHO Eastern Mediterranean Regional Office, Health Emergencies Department, Cairo, Egypt; 2 Health Emergencies Programme, World Health Organisation Regional Office for the Eastern Mediterranean, Cairo, Egypt; 3 WHE, World Health Organization, Geneve, Switzerland; 4 World Health Organization, Geneve, Switzerland

**Keywords:** COVID-19, Public Health, Control strategies, Health education and promotion, Health systems

**Summary box**

- Risk communication and community engagement (RCCE) is critical to the success of any public health preparedness and emergency response.
- Within the Eastern Mediterranean Region, most countries have limited RCCE capacity, with no formalised or institutionalised structures, processes or dedicated resources and mostly focused on the response.
- COVID-19 highlighted the importance of RCCE and need for systematic and sustainable investment.
- Lessons learnt and achievements made during COVID-19 present the opportunity for reinforcing for long-term RCCE system strengthening.

## Introduction

Risk communication and community engagement (RCCE) is critical to the success of any public health emergency preparedness and response.^1^ Risk communication is defined as a multilevel, multifaceted process which aims to help stakeholders define risks, identify hazards, assess vulnerabilities and promote community resilience.^2^ Community engagement is defined as a process of developing relationships that enable individuals, communities and organisations work together to address health-related issues and promote well-being to achieve positive health impact and outcomes.^3^ RCCE is defined as ‘the processes and approaches to systematically consult, engage, and communicate with communities who are at risk, or whose practices affect risk’.^4^ The effectiveness of any intervention is dependent on public trust and compliance with the specific public health interventions—both of which are key objectives of RCCE efforts.^2^ RCCE is one of the core capacities detailed in the International Health Regulations (IHR 2005), the global agreement that requires countries to build and sustain key capacities for national and global detection and response to public health threats.^5 6^

RCCE aims to encourage, enable and involve stakeholders in the prevention, preparedness, readiness and response to public health emergencies by adapting community-centred approaches.^7^ RCCE seeks to guide people to adopt risk-reducing practices and behaviours, as well enable community-led solutions for preventing and responding to the health and social impacts by empowering and enabling the active participation of individuals and communities. RCCE further aids in managing infodemics by promoting trust in health officials, strengthening social cohesion, and by providing clear, credible and accessible health advice that addresses communities’ perceptions, questions and concerns. This aids in reducing the spread and impact of misinformation, disinformation and uncertainty, which all can significantly undermine a response and threaten human health.^8^

In January 2020 following the detection and announcement of a new coronavirus called SARS-CoV-2, causing the disease COVID-19, WHO released a suite of RCCE recommendations for countries, building on the IHR (2005) RCCE capabilities, to prepare for and respond to COVID-19.^9^ Since these initial recommendations were released, the importance of applying RCCE principles and interventions across the spectrum of preparedness and response has been reinforced, in particular its role in helping stall transmission chains and increase public acceptance and adherence to public health and social measures (PHSM). This need has remained throughout the pandemic, as people’s behaviour and willingness to adhere to PHSM persist as one of the most powerful instrument for halting the virus’s spread.^8^

Within the WHO Eastern Mediterranean Region (the Region), there are over 664 million people living in 22 countries. Within this geographical area, there exists significant disparities that have been amplified by COVID-19.^10^ Approximately 101 million people require humanitarian assistance, representing 43% of the global humanitarian burden and 15% of the Region’s total population.^11^ Extreme vulnerability exist as a result of concurrent emergencies, with the Region having the largest number of children suffering from malnutrition. Health professionals, vaccines, drugs and medical supplies are all in short supply in already overburdened health systems. Lack of fuel, power and clean drinking water also contribute to the overall deterioration in quality healthcare delivery and protracted conflict in several countries has resulted in increased numbers of trauma care injuries and increased risk of disease outbreaks.^12^ As a result, the risk of rapid COVID-19 transmission in the region has been high, particularly in countries where refugees and internally displaced people live in overcrowded camp-like settings with inadequate sanitation, aggravated by fragile health systems, overburdened response capacities, and a suboptimal level of public health preparedness.^13^ As of 10 August 2022, more than 22 million cases of COVID-19 and 345 000 deaths were reported from the 22 countries in the Region.^14^

Between 2016 and 2019, the WHO Eastern Mediterranean Region Office (EMRO) supported countries to conduct Joint External Evaluations of their IHR (2005) capacities, including RCCE, followed by the development of national action plans for health security to address the identified gaps and challenges. The implementation of these plans was varied across countries in the Region, and most notably limited in relation to strengthening their RCCE capacities. In early 2020, in an attempt to enhancing preparedness and response to the pandemic, EMRO supported countries by providing a set of RCCE recommended actions to gear up readiness to the pandemic, protect population’s health and mitigate introduction and rapid transmission of the virus.^15^ All countries were supported with a templated RCCE action plan, and then assisted with technical guidance to contextualise this to their country situation and needs. In response to the need for coordinated action in the Region, a Regional RCCE Guiding Framework for COVID-19 was developed jointly by WHO, IFRC and UNICEF.^4^ This framework set forth the coordinated, multisectoral approach to addressing the extensive and long-term primary effects of the COVID-19 epidemic, as well as the secondary sociopolitical and economic implications felt by every country in the Region. The framework further set forth a variety of mandates, policies and partner capacities that must be mobilised to support this objective, guided by human rights and evidence-based approaches enshrined in key international conventions and treaties.^4^

In response to the pandemic, countries in the Region adopted a range of policies and PHSM, including personal preventative measures (hand and respiratory hygiene measures and mask wearing), environmental measures (disinfection and ventilation), as well as physical distancing measures (banning of mass gatherings, school closure, movement and travel restrictions), which were communicated and reinforced through RCCE interventions.^16^ To date, COVID-19 vaccines are being distributed in all countries, with complimentary RCCE efforts underway to build public acceptance and continue to generate demand for vaccines. However, as the pandemic persists, increasing socioeconomic pressure is undermining public adherence to these measures, despite the continuing transmission of COVID-19 in many countries.^17^

Moving forward, countries need to adopt RCCE adjusted interventions that aid in preventing or reducing transmission, taking into consideration living with COVID-19 for the foreseeable future.^18^ The initial requirement to build awareness and behaviours around COVID-19 has given way to the necessity to sustain behaviours and, eventually, to learn to live with the disease in the future.^4 8^ As a result, RCCE initiatives must go beyond simple information sharing, to encompass broader social participation efforts that empower communities to be actively engaged in the decision-making to health emergency preparedness and response.

To better understand countries’ RCCE capacity, and how capacity gained through COVID-19 can be sustained for broader public health emergency preparedness, an assessment of the planning, initiation, and implementation of RCCE efforts, including COVID-19 vaccine demand generation/RCCE related efforts, was undertaken. This assessment was conducted at different time points via on-site meetings and teleconferencing.

## RCCE country capacity assessment

The findings of this report are based on analysis from various WHO supported assessments conducted between 2016 to 2021. While the assessments took place over different periods of time, they nevertheless provide very similar findings that reinforce the points outlined in following section.

Between 2016 and 2019, 18 countries in the Region were supported by WHO to conducted IHR JEE (Afghanistan, Bahrain, Djibouti, Egypt, Iraq, Jordan, Kuwait, Lebanon, Libya, Morocco, Oman, Pakistan, Qatar, Saudi Arabia, Somalia, Sudan, Syria, Tunisia and United Arab Emirates).^19^ Within the RCCE assessment category, RCCE subcategories were assessed: risk communication systems, internal and partners coordination, public communication, rumour management and community engagement.^19^ Following the announcement of COVID-19, between March and June 2020 assessments of all countries’ RCCE readiness to respond were conducted through a mix of country visits and virtual meetings. The assessment checklist used included capacity building, in addition to subcategories detailed under the JEE.^19 20^ Between July 2020 and July 2022, intraaction reviews of countries’ COVID-19 response were also conducted in 14 countries (Afghanistan, Djibouti, Egypt, Jordan, Iraq, Islamic Republic of Iran, Lebanon, Libya, Pakistan, Saudi Arabia, Somalia, Sudan, Syria and Tunisia).^20 21^ In response to global vaccine distribution, between January and June 2021 WHO and UNICEF jointly conducted an online survey to assess 16 countries’ RCCE capacity for COVID-19 vaccine demand generation.

### RCCE capacity assessment in the Region

This paper provides an overview of regional RCCE assessments findings of countries’ preparedness and readiness to respond to COVID-19 and other emergencies.

1. *RCCE system and national plans:* in most countries, there is a lack of dedicated RCCE unit, and limited clarity regarding the roles and responsibilities of different national units who supported RCCE related interventions, including health promotion, health education and community-based initiatives. As a result, duplication of efforts and resources is common. In most countries, RCCE is a reactive, rather than proactive intervention, only activated during emergencies. Many countries have no national RCCE preparedness and response plans, although many developed COVID-19 specific RCCE response plans. These plans mainly rely on ad hoc systems established to communicate risks with vulnerable populations during public health emergencies. They often lack strategic approach and focus principally on information sharing via Information, Education and Communication (IEC) product development. A few countries have national all-hazard and multisectoral public health emergency RCCE plans that are formally endorsed.
2. *Internal and partner communication and coordination*: in general, there is a lack of formal and continuing multisectoral RCCE coordination mechanisms. In some countries, establishing multistakeholder communication coordination platforms and rolling out campaigns through decentralised structures has taken place as components of national health and development programmes. For COVID-19 preparedness and response, most countries in the Region have established RCCE coordination mechanisms, sometimes led by government with coleadership support from UNICEF and WHO or part of the health cluster coordination mechanism in some countries. However, integration of RCCE within the incident management and emergency operations systems, including public health emergency operation centre structure, is almost nonexistent. As a result, RCCE interventions are not well integrated into overall COVID-19 response efforts and opportunities are missed for social and behavioural insights to be used to inform other operational response areas, such as surveillance and case management. Furthermore, fragmented, and dual governance systems in countries experiencing conflict have led to difficulties or disruption in partner communication and coordination.
3. *Public communication including online platforms*: many countries have media/public relations departments that are responsible for planning and managing media relations during health emergencies. Spokespersons are identified at different levels but sometimes lacking relevant expertise and skills. IEC materials developed and adapted for vulnerable groups have been widely disseminated through official channels and active partners. Piloting and pretesting processes for messaging and communication material are limited. Many countries have a process in place for the timely dissemination of regular updates. However, access to multilingual communication products for migrant and refugee hosting in most countries is limited. Key media outlets in some countries have been identified and engaged, while this has been challenging in countries with political divisions. In the absence of a media strategy for public health emergencies in most countries, activities are typically conducted on an ad hoc basis and not informed by actual needs of different individuals and communities.
4. *Social listening and infodemic management*: online (traditional and social media channels) and offline (such as hotlines, community workers, studies, communities) platforms exist in most countries for social listening and community feedback; however, the majority of countries lack a systematic approach for collecting and analysing social and behavioural insights, integrating these data or using it to public health interventions.^8^ Few countries had established consistent practices and processes for addressing issues detected via social listening, such as rumours and misinformation, including via official press conferences or Ministry of Health communication channels. These consistent approaches to collecting and responding to social and behavioural insights significantly helped in building trust with communities and managing the infodemic.
5. *Community engagement*: Successful community engagement and social mobilisation campaigns are evident in almost all countries. These were intensified during the pandemic, with the majority of countries initiating rapid on-the-ground engagement and capacity-building using key influencers, including community and religious leaders, for outreach at community and household levels. Engagement of other sectors, civil society organisations and non-governmental organisations has also been accelerated in the response to COVID-19. There are trained community health workers, volunteers, and mobile health staff, including local responders such as Red Cross/Red Crescent societies and Scouts. However, two-way community engagement and accountability to affected populations is often absent. Communities have been seen as recipients of interventions rather than engaged in the discussion, planning, designing and implementation of public health interventions.
6. *Capacity building*: while many global and region resources are available, language has been a major barrier in upskilling countries in the Region. In response, regional and national partners developed multiple RCCE capacity building resources that were then rolled out intensively across the region. During lockdowns, banning of gatherings and restrictions imposed on international travel, capacity building activities were mostly conducted virtually. Training of leaders, influencers, community workers and media professionals were also facilitated. Although training was conducted, strategic capacity building planning and programming is lacking, and activities are mostly reactive, thereby insufficient. Identifying and training RCCE surge staff has also been a challenge, especially Arabic speaking professionals. Few countries managed to engage with academia for capacity building and support.
7. *COVID-19 vaccine planning and roll out*: RCCE was not well addressed in National Vaccination and Deployment Plans. While countries accelerated their efforts to receive and roll out COVID-19 vaccines, important issues such as building public acceptance and confidence in vaccines was not appropriately addressed, in part because of a fear of generating demand that exceeded supply capacity. This added to slow engagement and uptake for vaccines, and in some cases fuelled vaccine hesitancy by lack of engagement. In some countries, recent surveys identified upwards of 50% of the population expressed hesitancy to receiving a COVID-19 vaccine.
8. *Availability and use of data to generate evidence*: this area was not specifically targeted as part of the assessment; however, multiple assessments identified common findings. In almost all countries, ministries of health or partners conducted studies to collect social and behavioural insights. Overall, there are few examples of comprehensive collection, analysis and use of data to generate evidence and inform RCCE or other response interventions. While most countries use some form of evidence, there is a wide variation and inconsistency in the type and source of evidence used, commissioned or leveraged. In general, a systematic approach to understanding risk perception, knowledge, attitude and behaviour of target audiences has not been included in RCCE planning or intervention design. Research, monitoring and evaluation-related capacities in countries varied and are sometimes weak. When available data have been generated, challenges persist in the analysis and utilisation of these data.

## Lessons learned and areas for investment

Throughout the twenty-first century’s major public health events—including disease outbreaks of the SARS-CoV, the Middle East respiratory syndrome coronavirus, influenza A (H1N1), Ebola virus disease, other natural, biological and chemical events, and now monkeypox—RCCE has been a key indicator of success in the preparedness and response.^22^ In the case of COVID-19 and other future health emergencies, RCCE must integrate and coordinate across the other operational areas, including leadership and coordination, epidemiology, surveillance, contact tracing, rapid response systems, logistics and supply management, points of entry, referral facilities, case management, and infection prevention and control measures.^23^ In the East Mediterranean Region, the response to COVID-19 was exacerbated by the disparity in the impact of the pandemic’s primary (COVID-19-specific) and secondary (non-COVID-19) impacts. Moving forward, and taking into consideration the context of these countries, a Regional RCCE framework is needed to systematically promote and guide RCCE capacity building at national and subnational levels.^4^

## Community engagement

While several countries were able to minimise COVID-19 infection rates early, many were quickly overwhelmed. Reasons for the disparities are complicated; however, response efficacy is determined in part by the pace and scale of governmental engagement, as well as how communities participate, receive, perceive and respond to information and interventions.^24^ COVID-19 response necessitates consistent and coherent community engagement interventions. Public health practitioners must consider not only the evolving evidence about the pathogen and epidemiology, but also the sociocultural context and impact the event is having on the affected population. Most in-person community engagement was halted due to COVID-19 restrictions or was redirected to online engagement. In the future, feasibility of mid-media and in-person methods need to be reassessed, in particular where interpersonal communications and engagement are the main trusted approaches. This is particularly relevant for vulnerable populations living in camps, settlements or populations on the move.^25^ Engagement efforts must continue to promote adherence to behaviours and adaption of environments to comply with PHSM.

## Gender balance in RCCE leadership and planning

Women are typically under-represented in traditional communication channels, however should be mobilised through RCCE as change-makers and leaders for their communities.^4^ Despite the body of global evidence highlighting how gender balance results in stronger governance and more socially conscious decision-making, the impact of women as mothers, wives, trusted community members has been underplayed. Female leadership styles are often more oriented towards social welfare and collective well-being, as well as collaborative and participatory approaches to decision-making. These characteristics were evidenced in various female leaders during the COVID-19 pandemic, and should be harnessed and strengthened in future efforts by expanding the capacity and empowering women as community leaders for preparedness and response to future health emergencies.^26^

## Social listening and infodemic management

The pandemic exemplifies the essential influence of the world’s new information environment. Transmission of misinformation, disinformation and rumours has had significant impact on people’s behaviour and undermine overall responses. In response to these changing dynamics, models for forecasting virus spread or occurrence of other public health threats need to account for the population’s behavioural reaction to public health initiatives, as well as the communication dynamics behind content consumption.^27^ There is a critical need to accommodate social media in public communications strategies for listening, information sharing, and addressing rumours. In the Region, while traditional and social media use by populations is high, health literacy is weak. Monitoring and listening to online conversations, analysing patterns and sentiments, including rumours or misinformation, are all part of infodemic management and are an area for critical strengthening.^28^

## Conclusion

Regional analysis of the various national assessments identified that on average RCCE capacities were insufficient to respond to COVID-19, with the 2021 State Party Annual Reports providing a regional average capacity score of 60% (Median of 60%). Furthermore within the Region, there is significant disparity with country scores ranging from 20% in Somalia, to 100% in United Arab Emirates and Qatar.^29^

Trust and respect, as well as ownership and mutual accountability, will have an opportunity to flourish as communities become more directly engaged and involved in decision-making and actions. Governments and partners must work together to develop, monitor and review the implementation of national RCCE plans for preparedness and response to health emergencies and use lessons learnt to improve its impact and outcomes. Given the contextual similarities and challenges in RCCE capacities and approaches in the Region, countries must learn from one another, and exchange lessons learnt and best practices to fast-track RCCE achievements.

Building on learnings from the pandemic and previous assessments, countries in the Region are recommended to strategically map and plan with partners, invest into strengthening coordination structures, increase public trust through effective leadership, utilisation of social and behavioural insights and community participation, increase gender balance in RCCE leadership and document RCCE lessons learnt and best practices.

## Data Availability

All data relevant to the study are included in the article or uploaded as supplementary information.
